# Eyetracking Metrics in Young Onset Alzheimer’s Disease: A Window into Cognitive Visual Functions

**DOI:** 10.3389/fneur.2017.00377

**Published:** 2017-08-07

**Authors:** Ivanna M. Pavisic, Nicholas C. Firth, Samuel Parsons, David Martinez Rego, Timothy J. Shakespeare, Keir X. X. Yong, Catherine F. Slattery, Ross W. Paterson, Alexander J. M. Foulkes, Kirsty Macpherson, Amelia M. Carton, Daniel C. Alexander, John Shawe-Taylor, Nick C. Fox, Jonathan M. Schott, Sebastian J. Crutch, Silvia Primativo

**Affiliations:** ^1^Dementia Research Centre, Department of Neurodegenerative Diseases, Institute of Neurology, University College London, London, United Kingdom; ^2^Centre for Medical Image Computing, Department of Computer Science, University College London, London, United Kingdom; ^3^Centre for Computational Statistics and Machine Learning, Faculty of Engineering Science, Department of Computer Science, University College London, London, United Kingdom

**Keywords:** young onset Alzheimer’s disease, eye movements, eyetracking metrics, cognitive visual functions, machine learning, classification model

## Abstract

Young onset Alzheimer’s disease (YOAD) is defined as symptom onset before the age of 65 years and is particularly associated with phenotypic heterogeneity. Atypical presentations, such as the clinic-radiological visual syndrome posterior cortical atrophy (PCA), often lead to delays in accurate diagnosis. Eyetracking has been used to demonstrate basic oculomotor impairments in individuals with dementia. In the present study, we aim to explore the relationship between eyetracking metrics and standard tests of visual cognition in individuals with YOAD. Fifty-seven participants were included: 36 individuals with YOAD (*n* = 26 typical AD; *n* = 10 PCA) and 21 age-matched healthy controls. Participants completed three eyetracking experiments: fixation, pro-saccade, and smooth pursuit tasks. Summary metrics were used as outcome measures and their predictive value explored looking at correlations with visuoperceptual and visuospatial metrics. Significant correlations between eyetracking metrics and standard visual cognitive estimates are reported. A machine-learning approach using a classification method based on the smooth pursuit raw eyetracking data discriminates with approximately 95% accuracy patients and controls in cross-validation tests. Results suggest that the eyetracking paradigms of a relatively simple and specific nature provide measures not only reflecting basic oculomotor characteristics but also predicting higher order visuospatial and visuoperceptual impairments. Eyetracking measures can represent extremely useful markers during the diagnostic phase and may be exploited as potential outcome measures for clinical trials.

## Introduction

Alzheimer’s disease (AD) is the most common major neurodegenerative dementia type (1). While characterized by gradual and progressive episodic memory impairment, it is also associated with other cognitive impairments such as executive dysfunction, language, praxis, and complex visual processing deficits (2–5). Several phase three clinical trials have recently failed and there are no disease-modifying treatments available for AD (6, 7). Sensitive and sensible markers are needed to facilitate earlier diagnosis and to serve as outcome measures in clinical trials.

The focus of neuropsychological investigations in AD has previously been directed toward the study of anterograde episodic memory, and attentional and executive processes as primary consequences of AD and sources of functional impairment (8); by contrast, cognitive visual impairment has been widely overlooked (9). More recently, the presence of both low and high-level visual processing impairments has received more attention [for a review see Ref. (10)]. Deficits related to both the ventral and dorsal processing pathways have been described; impairments in object and facial recognition and color and pattern processing have been reported (11–13); abnormal performance has also been shown for tasks investigating visuospatial processing and motion perception (13–15). The presence of visual impairments has been associated with the severity of the disease (11, 13, 16, 17), leading to the possibility that visual testing could provide a method of screening and tracking AD. Studies suggest that cognitive visual deficits are more marked in young vs. late onset AD (18) and there may also be qualitative differences in the nature of the deficits. While some studies have highlighted the prominence of both ventral and dorsal stream deficits in late onset AD (13, 19), disproportionately impaired visuospatial ability has been described in young onset AD (18). Furthermore, the so called “visual variant” of AD—posterior cortical atrophy (PCA)—in which visual symptoms predominate (20–23) exhibits commonalities in cortical thinning with typical, amnestic AD particularly within temporoparietal regions (24), suggesting a continuum of visual impairment between typical AD (tAD) and PCA.

Posterior cortical atrophy is a progressive neurodegenerative syndrome mainly caused by AD pathology and characterized by progressive visuospatial and visuoperceptual dysfunction with relatively preserved memory, insight, and judgment (20). Individuals with PCA often manifest some or all of the features of Balint’s syndrome such as simultanagnosia, oculomotor apraxia, optic ataxia, and environmental agnosia (21–23, 25–28). Not only differences but also similarities have been described between PCA and tAD in terms of visual processing deficits (29), emphasizing the need for further study on the cognitive visual deficits in AD.

Recently, eyetracking technology has become more widely available and the simplicity of the instruments needed to collect good quality eyetracking data has enabled the application of the methodology to clinical populations (29–32). The resulting literature has demonstrated the presence of oculomotor impairment in AD patients as compared to age-matched healthy controls: longer saccade latencies in pro-saccade tasks and lower accuracy than controls in anti-saccade tasks have been reported in AD (33–37), together with abnormalities in saccadic accuracy (38–41).

The study by Shakespeare et al. (29) represents, to our knowledge, the only direct comparison between the basic oculomotor characteristics of individuals with PCA, tAD, and healthy controls. PCA patients showed increased time to saccadic target fixation, increased first major saccade latency, and decreased saccadic amplitude as compared to both tAD and controls. The patients with PCA also showed more frequent large intrusive saccades and lower longest period of fixation in the fixation stability task and lower pursuit and more saccades per trial in the smooth pursuit task as compared to controls. The authors also described impaired performance in the fixation stability test in tAD patients, who produced a large proportion of square wave jerks as compared to controls and individuals with PCA. As in PCA, tAD patients also had shorter maximum fixation period than the healthy controls. In the smooth pursuit task, both PCA and tAD patients showed significantly lower gain than the control group (29).

In cognitive psychology, eyetracking metrics have frequently been used to study higher order cognitive functions (42, 43). However, few studies in the field of dementia have utilized this potential to date. Crutcher and colleagues (44) and Richmond and colleagues (45) used a visual paired-comparison task and showed that eye movement metrics, such as number of fixations and fixation duration, can be indicative of short-term memory difficulties in a group of patients with mild cognitive impairment (MCI) as compared to age-matched controls. Fernández et al. (46) have investigated the semantic, working, and retrieval memory deficits in individuals with young onset AD by looking at differences with controls when reading high- and low-predictability sentences.

Despite the above-reported evidence of visual cognitive processing impairments and oculomotor deficits in both AD and PCA, little has been done so far to exploit eyetracking metrics as a route to explore deficits in visual cognition.

There are multiple potential advantages of using eyetracking metrics for studying visual cognitive processing in dementia. In contrast to many traditional neuropsychological assessments, eye movement recording does not require additional behavioral responses, such as button presses or verbal responses to make inferences about psychological changes. Eyetracking is also non-invasive and does not have contraindications, making it particularly well suited for patient studies. Moreover, modern eyetracking systems have excellent recording frames of up to 1,000 Hz, enabling the building of very large datasets (time series of *x* and *y* coordinates) in a relatively short amount of time (e.g., a 1,000 Hz system generates 600,000 *x*–*y* data coordinates for a 10-min recording session). Such qualities represent incentives to fully explore the possible contribution of eyetracking metrics to an accurate and sensitive diagnosis and as outcome markers for clinical trials. An increasingly practical approach to take advantage of the volume of eyetracking datasets involves the application of machine-learning methods, in which automatically generated feature vectors of individual participants may be used to assign categories to each participant.

In the present study, we explored the relationship between eyetracking and standard visual cognitive tests in individuals with young onset AD (both tAD and PCA). We extracted standard eyetracking summary metrics and used them to test the hypothesis according to which such metrics well correlate with visuoperceptual and visuospatial metrics derived from standard cognitive tests. We then applied a machine-learning approach to a proportion of the data to explore the possibility of automatically discriminating patients and healthy individuals on the basis of raw eyetracking metrics only (i.e., time series of *x* and *y* coordinates). Therefore, our secondary hypothesis was that machine-learning classifiers would offer the discriminative power (47) for the diagnosis of young onset AD among healthy controls based on oculomotor profiles during a discrete task.

## Materials and Methods

### Participants

The study was approved by the local Research Ethics Committee and all participants provided written informed consent according to guidelines established by the Declaration of Helsinki.

Data were collected from 36 individuals with young onset Alzheimer’s disease (YOAD) (26 patients with tAD and 10 patients with PCA) and 21 age-matched healthy controls. Patients with PCA fulfilled standard criteria for PCA (21, 22). Patients with AD had a clinical diagnosis of probable AD and fulfilled the NIA (National Institute of Aging) clinical criteria (5). Healthy controls and patients with YOAD did not differ in terms of age at assessment (two sample *t*-test, *t* = 0.09, *p* = 0.93) or years of education (Wilcoxon Mann–Whitney *U*-test, *z* = 1.57, *p* = 0.12) (Table 1). Healthy controls and YOAD patients differed in terms of Mini-Mental State Examination (MMSE) scores (Wilcoxon Mann–Whitney *U*-test, *z* = 6.21, *p* < 0.0001). Within the YOAD patients, tAD and PCA were matched in terms of disease duration [tAD: 5.0 (2.8) years and PCA: 5.6 (3.4) years, Wilcoxon Mann–Whitney *U*-test, *z* = −0.50, *p* = 0.62] and MMSE scores [tAD: 20.1 (0.8) and PCA: 23.1 (1.5), Wilcoxon Mann–Whitney *U*-test, *z* = −1.78, *p* = 0.07].

**Table 1:** Mean and SD demographic information and neuropsychology scores for the 36 patients with young onset Alzheimer’s disease (YOAD) and 21 age-matched healthy controls.

	Max score	Controls (*N* = 21)	YOAD (*N* = 36)	*N* (%) below 5%^a^	Normative mean (SD)
**Demographics**					
Gender M:F		11:10	17:19	NA	NA
Age (years)		61.0 (5.3)	60.9 (5.2)	NA	NA
Education (years)		16.5 (3.2)	15.3 (2.7)	NA	NA
Disease duration (years)		NA	5.2 (2.9)	NA	NA
**Background neuropsychology**					
MMSE	30	29.5 (0.7)	20.9 (4.4)	NA	29.0 (1.3)
Visual acuity: Snellen^b^	6/9	NA	6/9	NA	NA
WASI vocabulary	80	69.0 (8.5)	53.4 (18.3)	1 (2.8%)	NA
WASI matrices	32	26.7 (2.7)	8.1 (7.1)	8 (22.2%)	NA
Digit span forward (max)	8	7.3 (1.2)	5.4 (1.5)	11 (30.6%)	NA
Digit span backward (max)	7	5.4 (0.9)	3.2 (1.5)	10 (27.8%)	NA
RMT for faces	25	24.7 (0.8)	19.5 (4.4)	11 (30.6%)	22.8 (1.9)
RMT for words	25	24.4 (1.4)	17.5 (3.2)	27 (75.0%)	23.7 (1.8)
GDA: oral	24	13.8 (6.6)	2.9 (4.7)	25 (69.4%)	11.95 (5.1)
**Early visual processing**					
Shape detection (VOSP)	20	19.5 (0.8)	18.0 (1.4)	NA	19.5 (0.7)
**Visuoperceptual processing**					
Object decision (VOSP)	20	18.2 (1.4)	14.7 (3.9)	12 (33.3%)	17.7 (1.9)
Fragmented letters (VOSP)	20	19.5 (0.7)	11.2 (7.2)	23 (63.9%)	18.8 (1.4)
**Visuospatial processing**					
Dot counting: *n* correct	10	9.9 (0.3)	7.6 (2.9)	16 (44.4%)	9.9 (0.2)
A cancelation time (s)	90	21.1 (6.0)	54.0 (22.8)	27 (75.0%)	20.5 (6.5)
**Word recognition**					
NART: number of errors	50	11.5 (8.0)	20.1 (11.0)	NA	NA

*Mini-Mental State Examination (MMSE) (67); Cortical Vision Screening Test (CORVIST) (68); Wechsler Abbreviated Scale of Intelligence (WASI) (69); Wechsler Memory Scale Revised-digit span forwards and backwards (70); Matrices and Vocabulary subtest; Short Recognition Memory Test (RMT) for words and faces (71); oral Graded Difficulty Arithmetic (GDA) subtests addition and subtraction (72); Visual Object and Space Perception battery subtest object decision, shape detection, fragmented letters and dot counting (48); A cancelation (49); National Adult Reading Test (NART) (50). NA, not available*.

*^a^Percentage of scores below 5% percentile are shown for the YOAD group (the performance of controls did not reach this level)*.

*^b^Median value is reported*.

All participants had a detailed neuropsychology assessment investigating memory, language, executive function, and vision. The battery included six standard visual tasks, which were the focus of the subsequent correlational analysis between eyetracking and traditional neuropsychological metrics. Early visual processing was examined using the shape detection subtest from the Visual and Object Space Perception battery (VOSP) (48) where individuals were presented with 20 patterns, 11 of which contained a faint cross. Participants were asked to express a judgment as to whether a faint cross was present or not with score ranging from 10 (chance) to 20. Visuoperceptual processing was assessed using the fragmented letters and object decision VOSP subtests (48). In the former, individuals were asked to identify 20-fragmented capital letters presented one at a time with score ranging from 0 to 20. In the latter, they were asked to identify, among four silhouettes, which one represented a real life object [score ranged from 5 (chance) to 20]. Visuospatial processing was assessed using the dot-counting VOSP subtest (48) and letter cancelation test (49). In the dot-counting test, individuals were presented with 10 pages containing from 5 to 9 dots in different positions and asked to identify the number of dots without using their fingers (score range: 0–10). In the “A” cancelation task, participants were presented with an A4 sheet and asked to mark all the letters “A” embedded among 69 distractors (other letters) within 90 s. Last, single word recognition was assessed using the National Adult Reading Test (NART) (50) where individuals were asked to read 50 words aloud (score ranged from 0 to 50).

### Equipment

The experiment was run on a Dell 2120 desktop computer with a 23-inch screen at a viewing distance of 60 cm. Eye movements were recorded at 250 Hz using a head-mounted infrared video-based eye tracker (Eyelink II; SR Research). A chin rest was used to provide stability and maintain the viewing distance. The Eyelink system considered saccades using standard velocity and acceleration thresholds (30°/s and 8,000°/s^2^) and automatically identified periods with no saccadic movement as fixations. A 9-point calibration and validation were performed prior to each experiment. All the data were obtained from recordings with an average Cartesian prediction error of <1° during the validation. A drift correction procedure was used before each individual trial.

### Procedure

Three eyetracking experiments were performed:

*Fixation stability*: a red cross subtending at 0.5° of visual angle was presented in the center of the screen for 10 s. There was a practice trial followed by three test trials and participants were instructed to “look as closely as possible at the red cross without blinking for 10 s” (29, 51).
*Pro-saccade*: participants were initially presented with a black circle (subtending 0.4° of visual angle) having a white inner circle (0.1°) in the center of the screen lasting 500 ms. A blank screen was then displayed for 200 ms. After this, a target (black circle having a diameter of 0.75° and an inner white circle subtending 0.25°) was shown. The target remained on the screen until a fixation of minimum 250 ms duration was made within an area of 1.5° of visual angle from the center of the target (interest area) or after 5,000 ms from target onset. The participant’s task was to look at the target as quickly and accurately as possible when it appeared. The target appeared in one of 10 possible locations: 5°, 10°, or 15° either on the left or on the right, 5° or 10° either up or down. There were four trials for each location, giving a total of 40 test trials. Trials were split into two blocks (*n* = 20 each) with target locations randomized and balanced within each block. Four practice trials were used. The target positions were pseudo-randomized but their order was kept constant for all participants.
*Smooth pursuit*: the target was a red circle subtending 0.5° of visual angle in diameter. Twelve trials of target sinusoidal movement followed (horizontal: *n* = 6; vertical: *n* = 6). Two target velocities were used (10° and 20° of visual angle/second). The frequency of the target oscillation was set at 0.25 Hz for a target speed of 10°/s and 0.5 Hz for a target speed of 20°/s. Each trial lasted 10 s. The task started with two practice trials. Participants were instructed to follow the target as closely as possible with their eyes.

### Eyetracking Summary Metrics

All eyetracking recordings were visually inspected using Data Viewer and trials and/or participants were excluded if there was a significant signal loss that would have interfered with the data analysis and interpretation of results. Overall, 5.4% of the trials were excluded from the fixation stability task, 3.6% from the pro-saccade and 4% from the smooth pursuit tasks.

Blinks were identified and removed using Eyelink’s automated blink detection and practice trials were discarded from the analysis. Vision was binocular but eye movements from the right eye were recorded. If a problem was detected (i.e., poor eyesight, watery, or dry eye) recordings were performed using the left eye.

Statistical analyses were carried out using Stata (v. 12.1).

#### Fixation Stability

All participants performed the fixation stability task but two controls were excluded from analysis because of failure in signal detection. The relevant eyetracking metrics for the fixation stability task were large intrusive saccades, square wave jerks, and maximum fixation duration (29).

##### Number of Large Intrusive Saccades

The number of saccades with an amplitude greater than 2° of visual angle were identified as large intrusive saccades (29, 35).

##### Number of Square Wave Jerks

Square wave jerks were identified as saccades smaller than 2° in amplitude which took the gaze away from the target position, were followed within 300 ms by another saccade with a similar amplitude (difference in amplitude <0.75°) and took the gaze back to the target position (29, 52).

##### Maximum Fixation Duration

The longest period of fixation on the target (length of time between saccades) was measured for each participant (29, 35).

#### Pro-Saccade

All participants performed the pro-saccade task but two individuals (a YOAD patient and a control) were excluded from the analysis due to a failure in signal detection. For this task, the following variables were taken into account: accuracy, time to fixate the target, and number of saccades necessary to fixate the target. For these metrics, fixations made within an area of 1.5° from the center of the target (interest area) were considered to have met the target.

##### Accuracy

This metric was defined as the ability of the participant to fixate the target (within 1.5° from its center) while it was presented on the screen.

##### Time to Fixate the Target

The time between the target onset and the first fixation reaching the target was calculated. Negative values due to anticipatory saccades were either corrected if another fixation reaching the interest area was detected (0.09%) or removed if no fixation reached the interest area after target onset (0.14%).

##### Number of Saccades Necessary to Fixate the Target

The minimum number of saccades necessary to fixate the target was calculated for each trial.

#### Smooth Pursuit Task

All but three patients performed the smooth pursuit task. A control and a patient were excluded from the analysis due to a signal failure. The following variables were extracted: pursuit gain and proportion of time pursuing the target.

##### Pursuit Gain

This was defined by the ratio between the eye and the target velocity (in the relevant direction). Saccades and blinks were excluded and only a ratio greater than 0.5 was considered as pursuit gain. This cut-off was applied to dismiss eye movements happening after anticipatory saccades and turnaround points in each trial.

##### Proportion of Time Pursuing the Target

The proportion of time the participant spent pursuing the target during the trial was reported. This was calculated taking into account the number of samples considered as pursuit gain and multiplying this value by four (recordings were made at 250 Hz).

### Statistical Analysis

Differences in eyetracking metrics between the YOAD group and healthy controls were evaluated using linear regression models (clustered by participants) with robust SEs adjusted for repeated measures. Gender and age were considered as covariates for all metrics and stimulus distance and direction were considered as additional covariates for the pro-saccade task as well as target direction and velocity for the smooth pursuit task.

Normal distribution was assessed using the Shapiro–Wilk normality test. As the data were not normally distributed, a non-parametric measure of correlation (Spearman’s correlations) was performed and the coefficients reported. Correlations were explored between all oculomotor metrics and six standard visual cognitive tests including measures of early visual processing (shape detection); visuoperceptual processing (fragmented letters and object decision); visuospatial processing (dot counting and “A” cancelation); and single word recognition tests (NART).

### Machine Learning Classification Model

A machine-learning classification model is presented here as a proof of concept. As the statistical model aims to model movements in gaze location, the smooth pursuit experiment provided the most suitable data. The fixation stability and saccade experiments are designed to elicit 0 and one-gaze movements, respectively, and as such, their data did not provide enough information to discriminate between diagnostic classes on the basis of gaze movements. For this reason, the data from the smooth pursuit task were used in the pilot automatic classification procedure for the present study.

The automated classification procedure used the eyetracking data from the smooth pursuit task and modeled the movements in gaze location as the target moved. The gaze movements of each individual were used along with the statistical model to automatically generate feature vectors. These feature vectors were then used in a classification procedure that could predict the diagnoses of unseen individuals. The procedure, therefore, consisted of three components: (a) a fitted statistical model of each individual’s data, (b) the generation of feature vectors for each individual *via* the fitted model, and (c) the classification of individuals *via* their feature vectors.

A hidden Markov model (HMM) (53) was the statistical model used. This considers movements in gaze location, and assumes that each gaze movement has an underlying “intended” movement direction. We have assumed that each gaze movement was a noisy application of one of the following possibilities: “no movement,” “left,” “right,” “up,” or “down.” The HMM is slightly non-standard, in that fixed transformations of each gaze movement are applied before they are passed to the sub-model associated with each underlying intended movement direction. Furthermore, knowledge of where the gaze “should” be moving to was incorporated *via* the location of the target at each time. Intended movement directions that were more aligned to the target direction were given a higher likelihood. This implemented the natural assumption that individuals would follow the target as long as they were able to.

If *Y*_1:_*_T_* denotes the gaze movements for an individual over the course of one trial, *U*_1:_*_T_* denotes the direction of the target from the current gaze location at each time, and *X*_1:_*_T_* denotes the (unknown) underlying intended movement directions, then

(1) $$\begin{array}{l} \mathrm{Pr}\text{(}Y_{1:T},U_{1:T}\text{|}X_{1:T},\theta )=\prod_{t=1}^{T}N(Y_{t}^{\left(X_{t}\right)}\text{|}\mu_{X_{t}},\Sigma_{X_{t}})f(U_{t}|X_{t})\\ Pr(X_{1:T}|\theta )=\pi_{1}(X_{1})\prod_{t=2}^{T}P(X_{t}|X_{t-1}) \end{array}$$

where $Y_{t}^{\left(X_{t}\right)}$ are the transformed data and $\theta =(\mu_{X_{t}},\Sigma_{X_{t}},\pi_{1},P)$ are the parameters of the model. *N*(|μ,Σ) indicates the normal distribution with mean μ and covariance matrix Σ, and *f*(*U_t_*|*X_t_*) are parameter-free logarithmic distributions that go to 0 as the target direction diverges from the intended direction *X_t_*.

The model could be fit to the data using the EM algorithm (54). For every trial in the experiment, we fit the model to the data for one control. The individual that could follow the dot most accurately was subjectively chosen.

Once a model for each trial in the smooth pursuit experiment had been fitted, this could be used to generate feature vectors for each individual. The feature vectors are composites, made as the sum of “Fisher” feature vectors from the fitted models for each trial. Fisher feature vectors were computed as the gradient vectors of the data log-likelihood, evaluated at the fitted parameter settings. If *Z*^(^*^j^*^)^ is the Fisher feature vector for an individual in trial *j, Y*_1:_*_T_* are the gaze movement data for that trial, and $\hat{\theta }$ is the fitted parameter vector, then

(2) $$Z^{\left(j\right)}=\nabla_{\theta }\log Pr(Y_{1:T}|\theta ){|}_{\theta =\hat{\theta }}$$

which is a vector with as many dimensions as the model has parameters. The sum over trials gave the un-normalized feature vectors for each individual:

(3) $$\hat{Z}=\sum_{j}Z^{\left(j\right)}$$

Once all un-normalized feature vectors for an experiment had been computed, they were normalized element-wise by their SDs (over individuals):

(4) $$Z_{i}=\frac{{\hat{Z}}_{i}}{std\{{\hat{Z}}_{t}\}}$$

where *Z_i_* and ${\tilde{Z}}_{i}$ are the *i*^th^ elements of the normalized and un-normalized feature vectors, respectively.

These feature vectors can be used in many classification algorithms. We chose to use logistic regression to classify individuals. As there are approximately as many individuals in the dataset as there are dimensions to the feature vectors, the logistic regression classifier required some regularization. We used the Bayesian methodology to regularize the classifier, placing a sparsity prior on the weights in the classifier. If *d*(*Z*) is the diagnosis of the patient associated with feature vector *Z* (with 0 meaning control and 1 meaning either tAD or PCA), and *w* are the weights of the model, then

(5) $$\begin{array}{l} Pr(d(Z)=1|w)=\sigma (Zw)\\ Pr(w|\alpha )=\prod_{i}N(w_{i}|0,\alpha_{i}^{-1}) \end{array}$$

where σ() is the sigmoid function, and α are the Bayesian hyper-parameters of the model.

The performance of the classifier was assessed through cross-validation tests. Leave-one-out, leave-two-out, and leave-half-out tests were performed. In each of these tests, the data was partitioned multiple times into training and test sets. The classifier was trained on the training sets, and its predictions for the test sets were compared to the true diagnoses. If the proportion of correct predictions for each group class was high, then the classifier had high predictive power on the data within the dataset.

## Results

### Eyetracking Summary Metrics

Mean and SD performance metrics for fixation stability, pro-saccade and smooth pursuit tasks are shown in Table 2.

**Table 2:** Mean and SD of fixation stability, pro-saccade, and smooth pursuit metrics for young onset Alzheimer’s disease (YOAD) patients and age-matched healthy controls.

		YOAD	Controls

		Mean (SD)	Mean (SD)
**Fixation stability**			
Number of large intrusive saccades		2.5 (4.3)*	0.7 (1.7)*
Number of square wave jerks		0.9 (1.6)	0.9 (1.6)
Maximum fixation duration (ms)		1,950.7 (1,352.8)*	2,908.5 (2,062.1)*
**Pro-saccade**			
Accuracy	Overall	0.85 (0.35)*	0.94 (0.24)*
	5°	0.90 (0.31)	0.96 (0.19)
	10°	0.84 (0.37)*	0.92 (0.27)*
	15°	0.81 (0.39)*	0.93 (0.26)*
	Up	0.86 (0.35)	0.90 (0.30)
	Down	0.86 (0.32)*	0.93 (0.22)*
	Right	0.83 (0.38)*	0.97 (0.18)*
	Left	0.86 (0.35)	0.93 (0.25)
Time taken to reach the target (ms)	Overall	538.7 (682.3)*	328.7 (329.8)*
	5°	437.1 (583.0)*	306.1 (366.6)*
	10°	613.8 (777.3)*	337.4 (333.4)*
	15°	609.0 (650.4)*	358.8 (222.9)*
	Up	537.8 (648.5)*	365.6 (468.4)*
	Down	518.5 (571.2)*	329.2 (163.4)*
	Right	591.0 (827.1)*	300.6 (180.7)*
	Left	501.9 (613.8)*	333.6 (413.8)*
Saccades made to reach the target	Overall	3.1 (2.3)*	2.1 (1.2)*
	5°	2.7 (1.9)*	2.0 (1.2)*
	10°	3.4 (2.7)*	2.1 (1.1)*
	15°	3.4 (2.4)*	2.2 (1.2)*
	Up	3.3 (2.4)*	2.3 (1.3)*
	Down	3.1 (2.5)*	2.1 (1.1)*
	Right	3.0 (2.1)*	2.1 (1.2)*
	Left	3.0 (2.2)*	2.0 (1.1)*
**Smooth pursuit**			
Pursuit gain	Overall	1.4 (0.4)	1.4 (0.4)
	10°/s	1.4 (0.3)	1.4 (0.4)
	20°/s	1.3 (0.4)	1.4 (0.4)
	Horizontal	1.3 (0.3)	1.3 (0.3)
	Vertical	1.5 (0.4)	1.4 (0.4)
Prop. of time pursuing the target	Overall	0.4 (0.2)*	0.6 (0.2)*
	10°/s	0.5 (0.2)*	0.6 (0.1)*
	20°/s	0.3 (0.2)*	0.5 (0.2)*
	Horizontal	0.5 (0.2)*	0.7 (0.1)*
	Vertical	0.3 (0.1)*	0.5 (0.1)*

Statistically significant differences are highlighted in blue and marked with an asterisk (*) (for specific *p* values see text).

#### Fixation Stability

**Figure 1:**
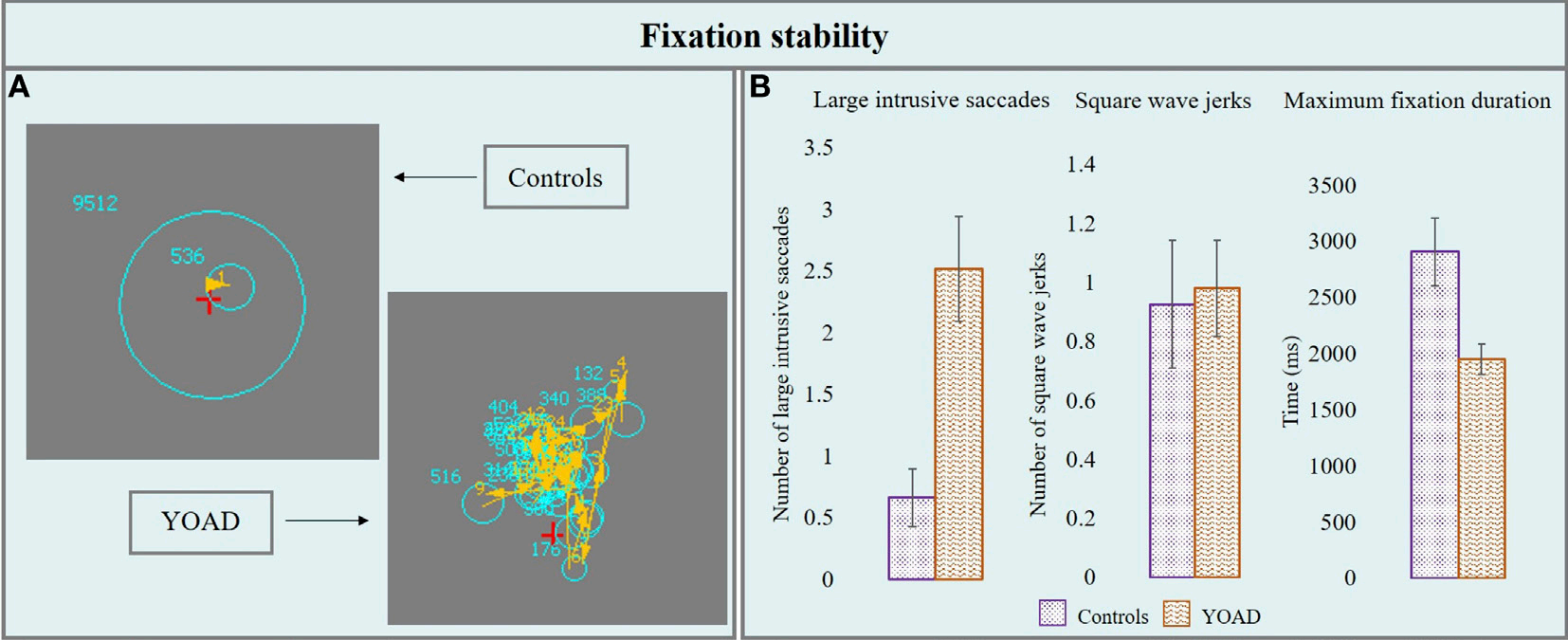
**(A)** Performance of a control and a young onset Alzheimer’s disease (YOAD) patient in the fixation stability task: light blue circles show fixations, yellow arrows indicate saccades, and red crosses represent target position. **(B)** Group means for controls and YOAD patients for the different task metrics. Error bars represent SE.

Results from the fixation stability task are represented in Figure 1 and in Table 2.

##### Number of Large Intrusive Saccades

Young onset Alzheimer’s disease patients made a statistically significant higher number of large intrusive saccades compared to healthy controls [YOAD: 2.5 (4.3), healthy controls: 0.7 (1.7), *t* = 2.5, *p* = 0.02].

##### Number of Square Wave Jerks

Healthy controls and YOAD patients did not show a statistically significant difference in terms of the average number of square wave jerks [YOAD: 0.9 (1.6); healthy controls 0.9 (1.6); *t* = 0.4, *p* = 0.60].

##### Maximum Fixation Duration

The longest period of fixation was significantly shorter for YOAD patients as compared to healthy controls [YOAD: 1,950.7 (1,352.8) ms; healthy controls: 2,908.5 (2,062.1) ms; *t* = −2.3, *p* = 0.02].

#### Pro-Saccade

**Figure 2:**
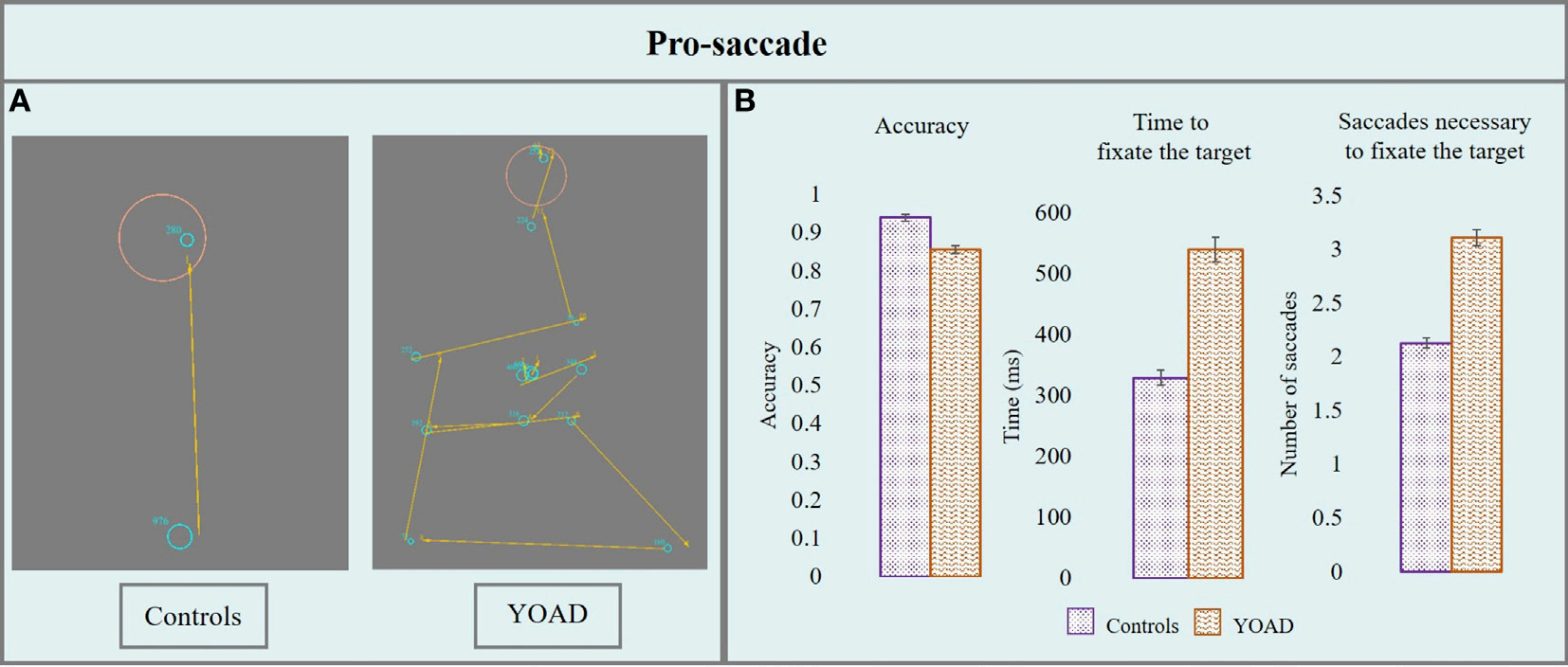
**(A)** Performance of a control and a patient with young onset Alzheimer’s disease (YOAD) in the pro-saccade task: light blue circles show fixations, yellow arrows indicate saccades, red crosses represent target position and orange circles outline the interest area (1.5° from the center of the target). **(B)** Group means for controls and YOAD patients for the different task metrics. Error bars represent standard error.

Results from the pro-saccade task are represented in Figure 2 and in Table 2.

##### Accuracy

Young onset Alzheimer’s disease patients had an overall significantly lower accuracy compared to healthy controls (*z* = −2.1, *p* = 0.04). The effect of stimulus distance was statistically significant: the greater the distance from the center, the lower the accuracy (*z* = −3.5, *p* < 0.001). When looking at the accuracy for specific stimulus distances YOAD patients showed a trend toward lower accuracy than controls at 10° and 15° (*z* = −1.81, *p* = 0.07) but not at 5° (*z* = −1.64, *p* = 0.10). The effect of stimulus direction was not statistically significant when comparing the accuracy of YOAD patients and healthy controls (*z* = 0.79, *p* = 0.40).

##### Time Taken to Fixate the Target

Young onset Alzheimer’s disease patients took significantly longer to fixate the target compared to healthy controls (*t* = 3.7, *p* = 0.001). The effect of the stimulus distance was significant on the time taken to fixate the target: the time increased with stimulus distance (*t* = 4.2, *p* < 0.001). A statistical trend was observed for the effect of stimulus direction (*t* = −1.9, *p* = 0.06). YOAD patients took more time to reach the target at all stimulus distances (all *p* < 0.01) as well as all stimulus directions (all *p* < 0.01) compared to controls.

##### Number of Saccades Necessary to Fixate the Target

Young onset Alzheimer’s disease patients made a statistically higher number of saccades in order to fixate the target (*t* = 3.65, *p* = 0.001). The effect of the stimulus distance (*t* = −3.7, *p* < 0.001) and the stimulus direction (*t* = 4.9, *p* < 0.001) both had a significant effect on the number of saccades necessary to reach the target. The number of saccades increased with stimulus distance and the greatest number of saccades for both groups was made when the stimulus moved upwards. YOAD patients made a greater number of saccades to reach the target for all stimulus distances (all *p* < 0.001) and stimulus directions (all *p* < 0.001) compared to controls.

#### Smooth Pursuit

**Figure 3:**
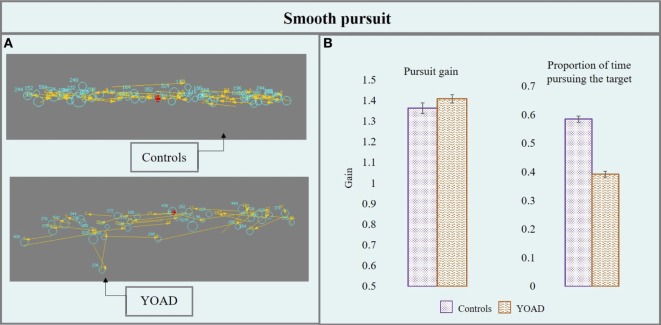
**(A)** Performance of a control and a young onset Alzheimer’s disease (YOAD) patient in the smooth pursuit task: light blue circles show fixations, yellow arrows indicate saccades, and red crosses represent target position. **(B)** Group means for controls and YOAD patients for the different task metrics. Error bars represent SE.

Results from the smooth pursuit task are represented in Figure 3 and are reported in Table 2.

##### Pursuit Gain

Young onset Alzheimer’s disease patients and healthy controls did not differ in terms of pursuit gain (*t* = 0.52, *p* = 0.60). Stimulus velocity did not have a statistically significant effect (*t* = −0.95, *p* = 0.30) but stimulus direction did (*t* = 4.06, *p* < 0.001). Pursuit gain was closer to one (one: eye velocity = target velocity, eyes moving at the exact target’s velocity) when the target moved horizontally (as opposed to vertically) for both groups.

##### Proportion of Time Pursuing the Target

Young onset Alzheimer’s disease patients spent significantly less time pursing the target compared to controls (*t* = −5.5, *p* < 0.001). Stimulus direction (*t* = −10.31, *p* < 0.001) and velocity (*t* = −10.84, *p* < 0.001) were both statistically significant: the proportion of time pursuing the target was greater when the target moved at 10°/s (as opposed to 20°/s) and horizontally (compared to vertically). YOAD patients spent less time pursuing the target when it was presented either horizontally (*t* = −4.29, *p* < 0.001) or vertically (*t* = −5.79, *p* < 0.001) as well as at both stimulus velocities (10°/s: *t* = −5.02, *p* < 0.001, 20°/s: *t* = −5.59, *p* < 0.001) when compared to controls.

#### Comparisons between Eyetracking Metrics in tAD and PCA Patients

Given the aims of the present study, all the described analyses were conducted by examining individuals with tAD and PCA as part of the same group of individuals with YOAD. There were statistically significant differences between the two groups of patients in only three out of the eight eyetracking metrics all of which showed a poorer performance of PCA compared to tAD: maximum fixation duration (controls: 2,908.5 ms, tAD: 2,183.6 ms, PCA: 1,342.4 ms, *t* = 2.85, *p* = 0.006); time to fixate the target (controls: 328.7 ms, tAD: 428.4 ms, PCA: 924.4 ms, *t* = 5.13, *p* < 0.001) and number of saccades necessary to reach the target (controls = 2.13, tAD:2.59, PCA: 4.86, *t* = 5.13, *p* < 0.001). PCA and tAD individual group performance was worse than that of healthy controls on all metrics for the three tasks (all *p* < 0.001).

No statistically significant differences were observed between tAD and PCA in the remaining five-eyetracking metrics. There was no difference in the number of square wave jerks (controls: 0.93, tAD: 1.04, PCA: 0.82, *t* = 0.11, *p* = 0.91) or pursuit gain (controls: 1.36, tAD: 1.35, PCA: 1.62, *t* = 1.50, *p* = 0.14) between tAD and PCA nor was there an effect of the phenotype in general (*t* = 0.36, *p* = 0.72 and *t* = 1.50, *p* = 0.14, respectively). No differences were observed between tAD and PCA in the number large intrusive saccades (controls: 0.67, tAD: 1.81, PCA: 4.39, *t* = −1.56, *p* = 0.12) and accuracy in the pro-saccade task (controls: 0.94, tAD: 0.89, PCA: 0.75, *z* = 1.55, *p* = 0.12). For these two metrics patients performed worse than controls but only PCA were statistically worse (*t* = 2.38, *p* = 0.02 and *z* = −2.62, *p* = 0.01, respectively). No difference was observed between the two groups of patients in the proportion of time pursuing the target (controls: 0.58, tAD: 0.40, PCA: 0.34, *t* = 1.11, *p* = 0.27) and the two performed statistically worse compared to controls (both *p* < 0.001).

### Relationship between Oculomotor Metrics and Standard Visual Cognitive Tests

In Table 3 coefficients and *p* values of correlations between estimates of visual cognitive processing and eyetracking metrics for the fixation stability, pro-saccade and smooth pursuit tasks are reported.

**Table 3:** Spearman’s rank coefficient (Spearman’s rho) and *p* values for correlations between visual cognitive tests and eyetracking metrics for the fixation stability, pro-saccade and smooth pursuit tasks.

		Eyetracking metrics							
		Fixation stability			Pro-saccade			Smooth pursuit	
		No. of large intrusive saccades	No. of square wave jerks	Max. fixation duration (ms)	Accuracy	Time to reach the target (ms)	Saccades made to fixate the target	Pursuit gain	Prop. of time pursuing the target
Visual Cognitive Test	VOSP shape detection	*r* = −0.16	*r* = 0.04	*r* = 0.19	*r* = −0.26	*r* = −0.19	*r* = −0.38	*r* = −0.09	*r* = 0.04
		*p* = 0.38	*p* = 0.81	*p* = 0.28	*p* = 0.14	*p* = 0.71	*p* = 0.03*	*p* = 0.61	*p* = 0.81
	VOSP object decision	*r* = −0.49	*r* = −0.32	*r* = 0.26	*r* = 0.09	*r* = −0.44	*r* = −0.64	*r* = −0.39	*r* = 0.29
		*p* = 0.003*	*p* = 0.06	*p* = 0.13	*p* = 0.61	*p* = 0.009*	*p* < 0.001*	*p* = 0.03*	*p* = 0.11
	VOSP fragmented letters	*r* = −0.41	*r* = −0.16	*r* = 0.26	*r* = 0.14	*r* = −0.40	*r* = −0.61	*r* = −0.23	*r* = 0.41
		*p* = 0.02*	*p* = 0.36	*p* = 0.14	*p* = 0.44	*p* = 0.02*	*p* < 0.001*	*p* = 0.22	*p* = 0.02*
	VOSP dot counting	*r* = −0.48	*r* = 0.07	*r* = 0.32	*r* = 0.18	*r* = −0.60	*r* = −0.54	*r* = −0.46	*r* = 0.66
		*p* = 0.005*	*p* = 0.70	*p* = 0.07	*p* = 0.32	*p* = 0.002*	*p* < 0.001*	*p* = 0.01*	*p* < 0.001*
	A cancelation time	*r* = 0.29	*r* = −0.15	*r* = −0.10	*r* = −0.04	*r* = 0.32	*r* = 0.45	*r* = 0.25	*r* = −0.49
		*p* = 0.10	*p* = 0.42	*p* = 0.57	*p* = 0.79	*p* = 0.07	*p* = 0.009*	*p* = 0.19	*p* = 0.006*
	National adult reading test	*r* = −0.23	*r* = −0.06	*r* = 0.14	*r* = 0.08	*r* = 0.18	*r* = −0.08	*r* = −0.14	*r* = 0.03
		*p* = 0.18	*p* = 0.72	*p* = 0.44	*p* = 0.64	*p* = 0.32	*p* = 0.66	*p* = 0.48	*p* = 0.88

Statistically significant correlations are highlighted in blue and their *p* values marked with an asterisk (*).

#### Fixation Stability

**Figure 4:**
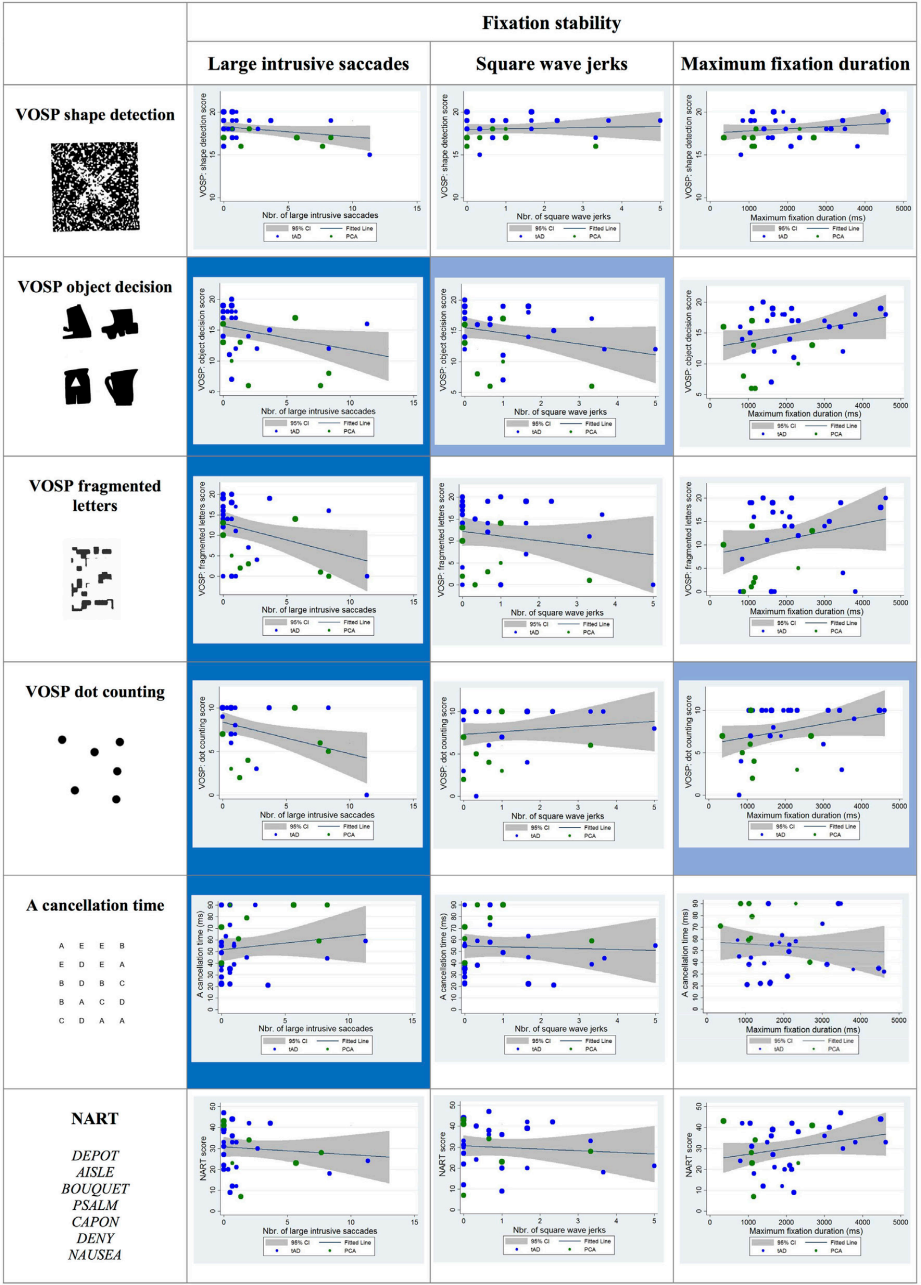
Scatter plots showing correlations between fixation stability metrics and visual cognitive scores for young onset Alzheimer’s disease patients. Statistically significant correlations are marked with a blue background and statistical trends (*p* > 0.05 and *p* < 0.10) in a lighter blue. Best fit line and 95% CI are shown. Each data point corresponds to a participant and its size is proportionate to the Mini-Mental State Examination score (indication of disease severity). typical AD (tAD) patients are shown in blue and posterior cortical atrophy (PCA) in green.

Statistically significant negative correlations were observed between the number of large intrusive saccades and the scores on the following visual cognitive tests: object decision, fragmented letter, and dot counting. The association between the number of square wave jerks and the score in the object decision test approached statistical significance (*p* = 0.06) as did the association between maximum fixation duration and dot-counting task (*p* = 0.07) (see Figure 4).

#### Pro-Saccade

Statistically significant negative correlations were reported between the time taken to fixate the target and the following visual cognitive tests: object decision, fragmented letters, and dot counting. The association between the time taken to fixate the target and the scores corresponding to the “A” cancelation task approached statistical significance (*p* = 0.07).

**Figure 5:**
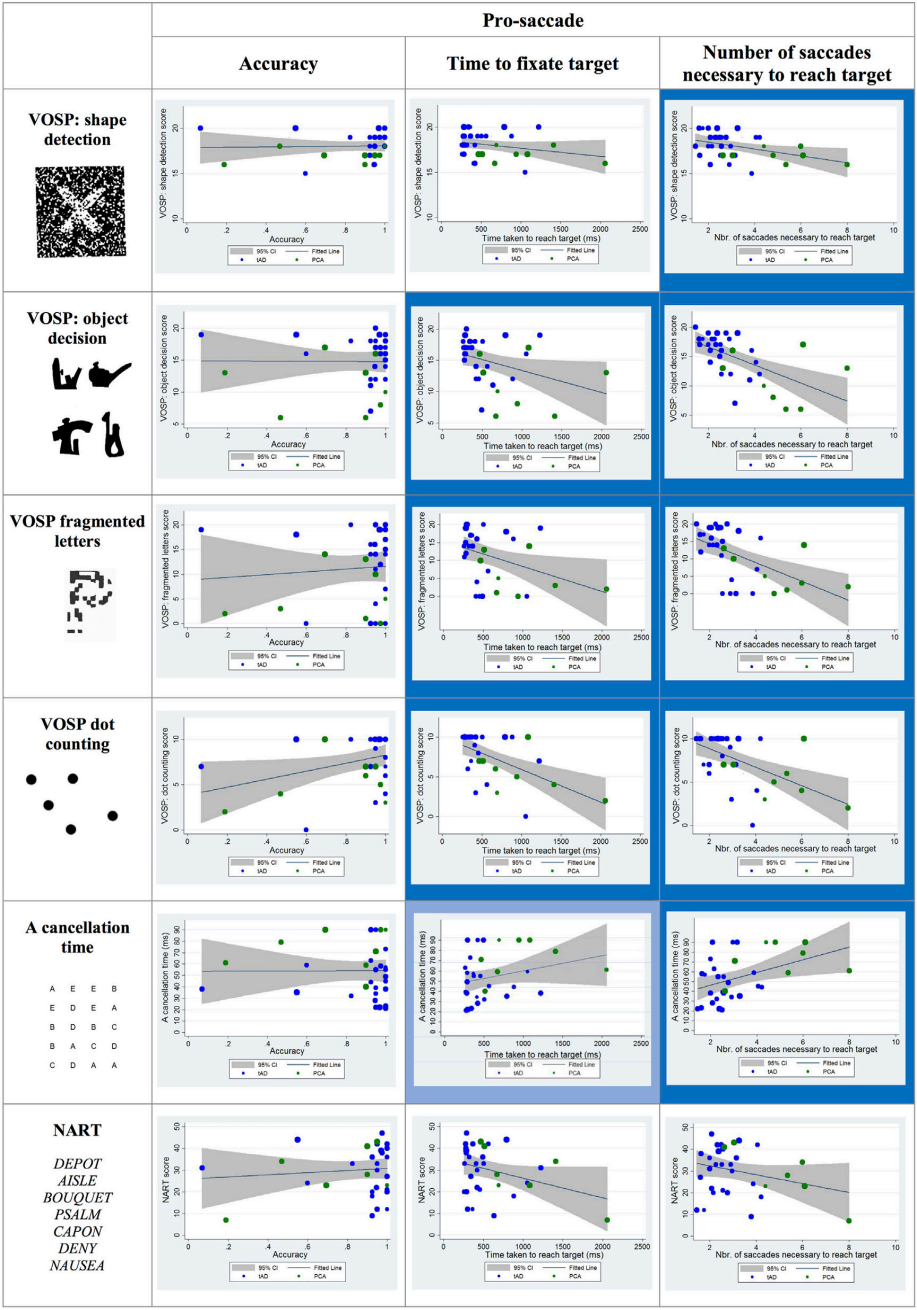
Scatter plots showing correlations between pro-saccade metrics and visual cognitive scores for young onset Alzheimer’s disease patients. Statistically significant correlations are marked with a blue background and statistical trends (*p* > 0.05 and *p* < 0.10) in a lighter blue. Best fit line and 95% CI are shown. Each data point corresponds to a participant and its size is proportionate to the Mini-Mental State Examination score (disease severity). typical AD (tAD) individuals are shown in blue and posterior cortical atrophy (PCA) in green.

Statistically significant negative correlations were found between the number of saccades necessary to fixate the target and the shape detection, object decision, fragmented letters, and dot-counting tests as well as a positive correlation with the “A” cancelation test scores (see Figure 5).

#### Smooth Pursuit

**Figure 6:**
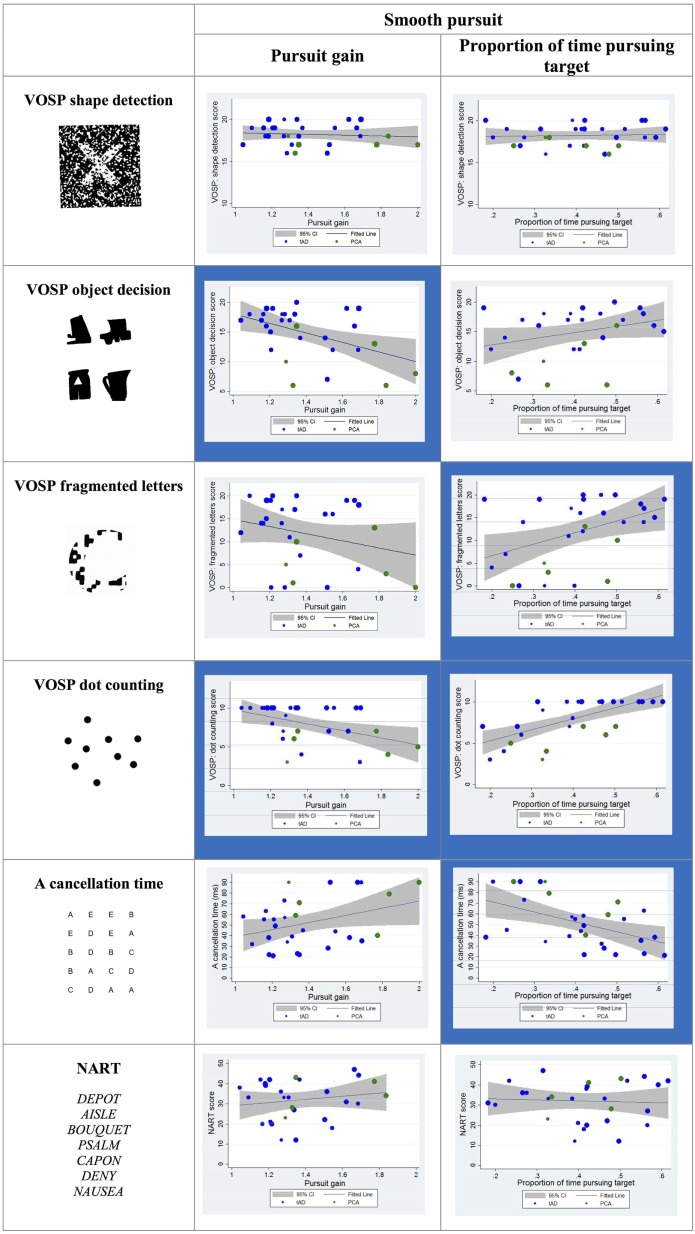
Scatter plots showing correlations between smooth pursuit metrics and visual cognitive scores for young onset Alzheimer’s disease patients. Statistically significant correlations are marked with a blue background and statistical trends (*p* > 0.05 and *p* < 0.10) in a lighter blue. Best fit line and 95% CI are shown. Each data point corresponds to a participant and its size is proportionate to the Mini-Mental State Examination score (disease severity). typical AD (tAD) individuals are shown in blue and posterior cortical atrophy (PCA) in green.

Statistically significant correlations were observed between visual cognitive scores and both the pursuit gain and the proportion of time spent pursuing the target during the trial. In particular, the pursuit gain scores for YOAD patients negatively correlated with the object decision and dot-counting tests. The proportion of time spent pursuing the target positively correlated with the fragmented letters, dot counting and negatively with the “A” cancelation test scores (see Figure 6).

### Machine-Learning Classification Model

Fitting the HMM to each trial of the smooth pursuit experiment resulted in fitted parameters that conformed to expectations. In particular, the fitted model placed significantly more likelihood on the movement directions that followed the target than on any other direction. The results of the logistic regression classifier using the automatically generated feature vectors were able to discriminate with 95% accuracy patients and controls. Feature vectors generated as described above are effective at separating the patients in the dataset into their diagnostic classes. The results of each test for the experiment are shown in Table 4. As can be seen, the predicted diagnoses show at least 95% accuracy for both diagnostic classes (patients vs. controls). While these results are very promising, they do only relate to the data from a relatively small number of individuals on a single test. An expanded experimental set-up, with a larger sample size, would be able to further verify the utility of this methodology for accurately predicting the diagnoses of previously unseen individuals.

**Table 4:** Table showing the results of cross-validation tests for the predictive power of the Bayesian logistic regression classifier.

Test	Actual diagnosis	Predicted diagnosis	
		Control	Young onset Alzheimer’s disease (YOAD) patient
L-1-O	Control	0.95	0.05
	YOAD patient	0.03	0.97
L-2-O	Control	0.95	0.05
	YOAD patient	0.03	0.97
L-H-O	Control	0.94	0.06
	YOAD patient	0.04	0.96

*Leave-one-out (L-1-O) tests take each individual in turn and train the classifier on all other individuals’ feature vectors. The diagnostic status of that individual is predicted by the classifier and compared to the actual diagnosis (YOAD patient vs. control). Leave-two-out (L-2-O) tests take each possible pair of one control individual and one patient, train on all other individuals, and then predict the diagnosis of the original pair. The leave-half-out (L-H-O) test takes 500 random partitions of the data, with half of each diagnostic class in each partition, trains on one partition and predicts the diagnoses of the other. The columns of the table represent predicted diagnostic classes, and the rows represent actual diagnoses*.

## Discussion

In the present study, we examined basic oculomotor metrics in individuals with YOAD and explored the link between such metrics and measures of visual cognition.

Results confirmed that patients have abnormal eye movement patterns in fixation stability, saccade and smooth pursuit tasks as compared to age-matched healthy controls. In the fixation stability task, YOAD patients showed a larger number of large intrusive saccades and shorter fixation duration. In the pro-saccade task, they were less accurate, required a longer amount of time and greater saccadic movements to fixate the target. Finally, in the smooth pursuit task, YOAD patients spent a shorter amount of time pursuing the target and made more interruptive saccades compared to controls. Results also demonstrated that these established basic control and movement metrics were significantly associated with measures of higher order visual cognition. In the fixation stability task the number of large intrusive saccades negatively correlated with performance in the object decision, fragmented letters and dot-counting tests. Pro-saccade metrics such as the time taken to reach the target and the number of saccades made in the process negatively correlated with object decision, fragmented letters, and dot-counting test scores. Additionally the “number of saccades necessary to reach the target” also correlated with shape detection scores and as the time taken to complete the “A” cancelation task. The eyetracking metrics extracted from the smooth pursuit task correlated with object decision, fragmented letters, dot counting and time to complete the “A” cancelation tests.

To our knowledge, this is the first time that the systematic impairment of basic oculomotor functions is reported in patients with young onset dementia as a single group and that the relationship between such impairment and visual cognition is explored.

Our data underline the extent of visual cognitive impairments in individuals with YOAD (18). Awareness of dementia-related visual dysfunction in tAD is increasing (11, 12, 55) but, especially in the early stages of the disease, sensitive measures are required in order to highlight subtle changes that can potentially be discriminated from normal aging. For example, a recent study has shown that an eyetracking behavioral task can predict the conversion from cognitively normal to MCI and from MCI to AD up to three years prior to a change in clinical diagnosis (56). The presence of both eye movement deficits and impairments in visual processing is in accordance with the neuroimaging and neuropathological literature showing that in AD multi-focal neuronal degeneration affects visual areas in the occipital, temporal and parietal lobes (57–59) and subcortical regions such as pulvinar (60) that process visual information and orient eye movements accordingly (61, 62).

The present study provides preliminary evidence suggesting the potential use of eyetracking metrics as markers of high-level vision and other cognitive domains. Examining the pattern of correlations, it should be noted that some eyetracking metrics, especially the “number of saccades needed to fixate the target,” correlated with most of the visual cognitive tests, possibly reflecting non-specific associations with disease severity or the ubiquity of pro-saccade generation deficits in YOAD patients. However, not all eyetracking metrics had such widespread associations. Particularly evident were the impact of “large intrusive saccades” and “time to reach a pro-saccade target” upon the visuoperceptual tests (object decision and fragmented letters) and the visuospatial dot-counting test. All three of these tests require scanning over relatively small visual areas and across multiple discrete perceptual items, all of which are relevant to the task demands. By contrast, neither of these eyetracking metrics was correlated significantly with the “A” cancelation test, in which visual attention must be deployed over a much wider visual area and across items, only a small proportion of which constitute task-relevant targets. Also possibly noteworthy are the significant correlations between the “proportion of time pursuing target” metric from the smooth pursuit task and the fragmented letter, dot counting and “A” cancelation tests. Unlike the other tests with which these correlations were not observed, these three tests all require participants to trace a specific continuous visual route through separated stimuli, whether that route pertains to the shape of a large letter (fragmented letter), the path through a group of dots that permits them to be counted (and not accidentally re-counted) in an efficient manner (dot counting), or the line-by-line orderly searching for target “A”s among other distractor letters (A cancelation).

Of equal note is the relative absence of correlations between eyetracking metrics and either shape detection or reading. In the case of shape detection, which was only correlated with the number of saccades required to reach the pro-saccade target, this may relate in part to the limited dynamic range of the test (all patient scores between 15 and 20 out of 20). However, for single word reading, with which there were no significant correlations and which with small print can be achieved in a single fixation, disordered eye movements such as large saccadic intrusions appear to have relatively little impact on accuracy (though note that reading latencies, if recorded, may have elicited a different result).

Naturally, such a qualitative examination of patterns of association has inherent limitations in determining causal relations between cognitive functions and observed behaviors. However, the current data arguably provide a useful starting point for generating testable hypotheses regarding the ability of certain eye movement patterns and paradigms to index specific cognitive abilities and deficits among dementia patients and other clinical populations.

Eyetracking-based measures of cognition may offer certain advantages over traditional pen and paper-based cognitive tests in some dementia contexts. Eyetracking data by definition do not suffer from ceiling and floor effects, which are instead common problems when exploring cognitive performance in patients (floor effect) and comparing it with performance in age-matched healthy controls (ceiling effect). Tasks such a fixation stability, saccade generation and smooth pursuit require minimal verbal instructions. Eye movement metrics derived from appropriate test designs may also be less vulnerable to the practice effects normally observed in standard cognitive testing, allowing for re-testing in the context of longitudinal assessments or before/after a trial phase.

One further potential advantage of eyetracking-based measures of visual cognition and other cognitive capacities is the type and scale of data generated. The large datasets that can be extracted in terms of time series of *x* and *y* coordinates open up new avenues of statistical analysis on individual trials. This may contribute to the design of shorter, less stressful cognitive assessments for patients. In the current study, we have provided proof of principle evidence for the feasibility of using an eyetracking dataset as the input for a machine-learning classification model to discriminate YOAD patients and controls on the basis of eye movements alone. While eyetracking in isolation is unlikely to ever be a primary determinant of clinical decision making, the application of a machine-learning approach to such examinations may add value in detecting change in at risk and presymptomatic individuals, monitoring progression over time (especially in the context of clinical trials), improving the discrimination and characterization disease and syndromic phenotypes.

The study had a number of potential limitations. First, the sample size was relatively small, and thus we have not been able to clarify whether the eye movement impairment is more widely a consequence of the disease severity rather than being a direct indication of visual cognition. Nonetheless, previous studies exploring eyetracking metrics in individuals affected by different types of dementia but matched for disease severity have shown that eye movement deficits can be disease-specific (33, 63). Second, the study did not include markers of focal and sustained attention, deficits in which may have contributed to both eyetracking metrics and visual cognition estimates. Third, we only found limited evidence of differences between PCA and tAD in terms of eyetracking metrics. This result can have several explanations. The PCA group size was very small and the individual variability within each group was very large, as is frequently described within this literature (28, 64, 65). This might reflect the biological reality of YOAD where a greater prevalence of visual deficits across the population has already been suggested (18) and/or the phenotypic continuum of visual impairment across the tAD-PCA spectrum (64, 66). To address further the issue of individual phenotypic differences, rather than a binary PCA/tAD diagnostic category, future studies involving a larger sample size should take into account the possibility of using a quantitative continuous metric of visual cognitive impairment, such as a ratio of memory to perceptual and/or spatial scores.

In conclusion, we have demonstrated that basic oculomotor metrics can provide information about not only the oculomotor system and its functionality *per se*, but also about high-level visual cognition. We have also shown that such metrics can be used in a machine-learning approach to discriminate between YOAD patients and healthy controls. Visual deficits represent a common feature in AD and eyetracking metrics may have potential as sensitive markers, particularly as outcome measures for clinical trials.

## Ethics Statement

The study was approved by the local Research Ethics Committee and all participants provided written informed consent according to guidelines established by the Declaration of Helsinki.

## Author Contributions

IP and SPrimativo: conception of the work, analysis and interpretation of data, drafting, final approval of the manuscript, and agreement to be accountable for all aspects of the work in ensuring that questions related to the accuracy or integrity of any part of the work are investigated and resolved. SParsons and DR: analysis of the data, drafting, final approval of the manuscript, and agreement to be accountable for all aspects of the work in ensuring that questions related to the accuracy or integrity of any part of the work are investigated and resolved. TS, CS, RP, KM, AC, KY, NFirth, AF, DA, JT, NFox, and JS: contribution to the conception and design of the study, collection of the data, revising it, final approval of the manuscript, and agreement to be accountable for all aspects of the work in ensuring that questions related to the accuracy or integrity of any part of the work are investigated and resolved. SC: conception of the work, interpretation of data, contributed to the drafting of the manuscript, final approval, and agreement to be accountable for all aspects of the work in ensuring that questions related to the accuracy or integrity of the work are investigated and resolved.

## Conflict of Interest Statement

The authors declare that the research was conducted in the absence of any commercial or financial relationships that could be construed as a potential conflict of interest.
